# Mesonephric Adenocarcinoma of Uterine Cervix: A Case Report and Review of the Literature

**DOI:** 10.30699/IJP.2020.125459.2375

**Published:** 2020-12-21

**Authors:** Fatemeh Nili, Samaneh Salarvand, Hana Saffar, Bita Kalaghchi, Reza Ghalehtaki

**Affiliations:** 1 *Department of Anatomical and Clinical Pathology, Cancer Institute, Tehran University of Medical Sciences, Tehran, Iran*; 2 *Radiation Oncology Research Center (RORC), Cancer Research Institute, Tehran University of Medical Sciences, Tehran, Iran*; 3 *Department of Radiation Oncology, Cancer Institute, Imam Khomeini Hospital Complex, Tehran University of Medical Sciences, Tehran, Iran*

**Keywords:** Adenocarcinoma, Gynecologic Neoplasms, Mesonephric Ducts, Rare Diseases, Uterine Cervical Neoplasms

## Abstract

Mesonephric adenocarcinoma of the uterine cervix is an extremely rare tumor of the female genital tract which derives from the remnants of embryonic mesonephric ducts and its prognosis, diagnosis and treatment is rather challenging. We report a case of a 46-year-old woman with history of abnormal uterine bleeding and an enlarged uterine cervix on physical examination without obvious mass lesion. She was clinically underdiagnosed with cervical myoma and mesonephric hyperplasia. After simple hysterectomy, stage IB2 mesonephric adenocarcinoma was diagnosed. Despite adjuvant chemoradiation, she presented with peritoneal and locoregional recurrence in less than a year. So, in the presence of abnormal bleeding and cervical mass, mesonephric hyperplasia in cervical biopsy specimen should be suspected for adenocarcinoma. Radical hysterectomy and complete staging with or without salpingo-oophorectomy is the mainstay of treatment. Despite all ambiguities, due to the small number of reported cases, the overall prognosis seems to be less favorable than conventional cervical adenocarcinoma.

## Introduction

Mesonephric adenocarcinoma of the uterine cervix is an extremely rare tumor of the female genital tract which derives from the remnants of embryonic mesonephric (Wolffian) ducts (1). These remnants may persist in the broad ligaments or the lateral walls of uterine cervix or vagina, appearing as small groups of glands or tubules. They may transform to benign cysts or hyperplasia and rarely to malignant tumors (2). To the best of our knowledge, only about 40 cases of mesonephric adenocarcinoma of cervix have been reported so far in the literature (3). 

Mesonephric adenocarcinoma usually shows a mixture of morphologic patterns making the pathologic diagnosis challenging. Another issue is that this entity may be confused with carcinomas of Mullerian origin like clear cell carcinoma (2,3).

Due to its rarity, the clinical features, prognosis, and the best treatment are still unclear. No consensus in therapeutic methods exist and different strategies may be applied for treatment. 

## Case Presentation

A 46-year-old gravid4, para2, abortion2 premenopausal woman with complaint of abnormal uterine bleeding, abdominal pain and discomfort was admitted to our hospital. Her past medical history was unremarkable except for two spontaneous abortions and two uneventful deliveries. She denied using any medical, herbal, or recreational drugs or smoking. The general physical exam was unremarkable. The gynecologic examination revealed an enlarged uterine cervix without mucosal ulceration or massive lesion. Cervical Papanicolaou (Pap) smear examination was normal. Transvaginal ultrasonography revealed a hypoechoic mass measuring 36×30 mm in uterine cervix with increased vascularity in color Doppler study. The endometrial thickness was 9 mm. Magnetic resonance imaging (MRI) showed an anterior cervical mass (40×30 mm) with evident mass effect on upper cervical canal. No extension was noted toward parametria, vagina and uterus body. There was a right, simple, 3-cm ovarian cyst as well. She underwent biopsy of the cervical mass. The light microscopic examination showed acute and chronic cervicitis and mesonephric hyperplasia present in the stroma. There was no pathologic evidence of malignancy in the specimen. The final clinical impression was uterine cervix myoma, so the patient underwent simple abdominal hysterectomy and bilateral salpingectomy without lymph node sampling/dissection. 

**Fig. 1:**
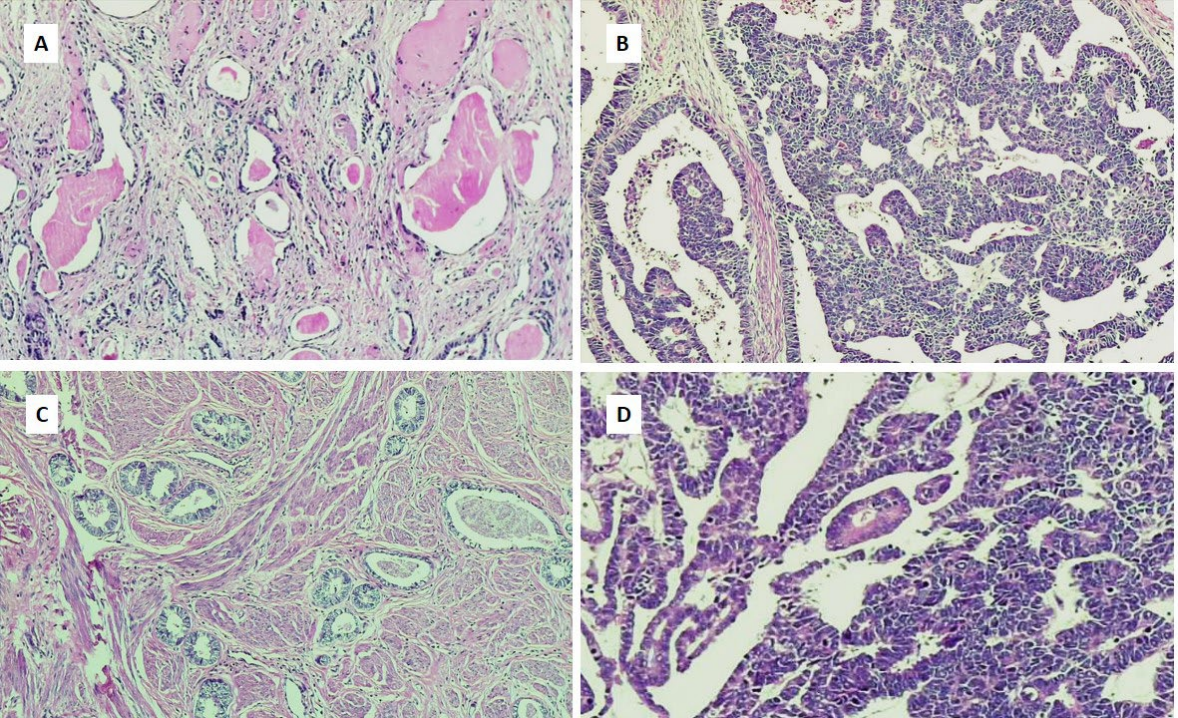
(A) Uterine cervix biopsy shows tubular structures with luminal eosinophilic secretions and flat to columnar epithelium compatible with florid mesonephric hyperplasia (×100). (B-D) Mesonephric adenocarcinoma of uterine cervix with papillary, solid, glandular, and tubular structures infiltrated in cervical stroma. Hematoxylin and eosin sections (×100, ×400)

**Fig. 2:**
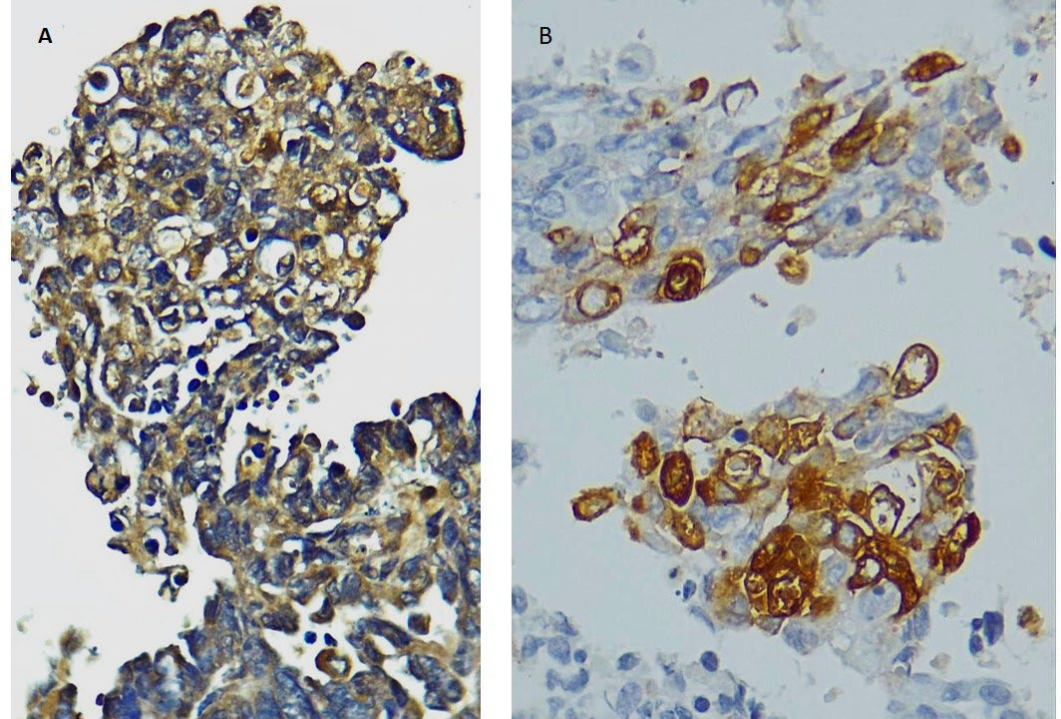
Immunohistochemical staining showing weak positive reaction for calretinin (A) and CD10 (B) in tumoral cells (×400).

On pathologic examination, a globally enlarged cervix was seen containing a 4.5×4×3 cm mass on cutting of cervical stroma. Microscopic examination showed an infiltration of neoplastic tissue composed of columnar cells with atypical hyperchromatic nuclei, arranged in glandular, tubular, and complex papillary structures which were deeply located in cervical stroma. At the periphery and near the mucosal surface, diffuse and florid proliferation of tubular structures with bland-looking cuboidal epithelium and eosinophilic luminal secretions were noted (Figure 1). The immunohistochemical (IHC) staining showed positive reaction of tumoral cells for Pan-cytokeratin (CK), Pax-8 and CK7. Vimentin, Calretinin, and CD10 were focally positive in tumoral cells as well as in hyperplastic tubules. There was a negative reactivity for CEA, WT1, estrogen and progesterone receptors (Figure 2). The final diagnosis was mesonephric adenocarcinoma, histologic grade 2 to 3. The depth of cervical stromal invasion was more than 2/3 of cervical wall. Lympho-vascular (LVI) and perineural invasions were also identified. Myometrium and vaginal cuff, fallopian tubes, parametria and surgical margins were free from tumor (International Federation of Obstetrics and Gynecologists stage IB2).

One month after the surgery, the patient was referred for adjuvant treatment. The consultant radiation oncologist and multidisciplinary team provided her with the option of post-op chemoradiotherapy due to size of the tumor (>4 cm), presence of deep stromal invasion (2 cm), LVI and incomplete surgery. Thus, the adjuvant treatment plan included external beam radiotherapy (50.4 Gray in 28 fractions) with concurrent chemotherapy (weekly intravenous cisplatin (35 mg/m^2^)) followed by vaginal cuff brachytherapy (12 Gy at 0.5 cm from mucosal surface divided in 2 fractions). 

The treatment was completed in 2 months as planned. Then the patient was visited regularly, every 3 months. Her follow up was unremarkable until 9 months after radiation when she complained of abdominal pain. Computed tomography (CT) scan imaging of the abdomen and pelvis, revealed multiple peritoneal masses with maximum diameter 69 mm at sigmoid and transverse mesocolon in favor of peritoneal and locoregional recurrence. In addition, a FDG-Positron emission tomography (PET) was performed, which revealed multiple lesions with high FDG uptake (hyper-metabolic) in pelvic cavity, liver and both lungs suggesting simultaneous locoregional and distant failure. In her last follow up, she was taking maintenance chemotherapy and her disease was stable. 

## Discussion

Remnants of embryologic mesonephric duct system are found in 22% of adult uterine cervices while this figure is 40% among newborns and children. They appear as small groups of glands or tubules, lined by single layers of cuboidal to columnar non-ciliated epithelium with scanty clear to slightly eosinophilic cytoplasm. The cells lack mucin (in contrast to endocervical epithelium) or glycogen. Tubular lumens usually contain an eosinophilic material. These remnants may transform to their benign (i.e., hyperplasia) or malignant counterparts (2,4). Lobular, diffuse, and ductal subgroups are three different types of mesonephric hyperplasia. Mesonephric adenocarcinoma is the most important differential diagnosis. It is a rare entity that usually presents with postmenopausal bleeding and cervical mass. Meanwhile, mesonephric hyperplasia is usually small (less than 25 mm) and asymptomatic. Nuclear atypia, conspicuous mitotic figures, solid growth pattern and ki67 index more than 15% are the other histologic features of the malignancy (5). There is slight chance that the tumor is detected on Pap smear (3). They are usually diagnosed on cervical biopsy, curettage, or hysterectomy specimens.

Malignant mesonephric tumors have been reported in uterine corpus, cervix, broad ligaments, urinary bladder, urethra and urethral diverticulum (6). Due to various morphologic patterns and similarity to other entities such as clear cell carcinoma, serous carcinoma, malignant mixed Mullerian tumor, and endometrioid adenocarcinoma, the incidence of mesonephric carcinoma is probably underestimated (2). Presence of florid mesonephric remnants and hyperplasia is a typical background in mesonephric adenocarcinoma (7). Immunohistochemical staining may be helpful in differentiating it from Mullerian counterparts (8). Positive staining of tumoral cells for CD10, EMA, PAX-8, Vimentin and Calretinin along with negative reaction for CEA, ER and PR is suggestive of mesonephric adenocarcinoma (9). However, none of them are specific markers for mesonephric structures, except for CD10 which is considered to be a good marker of mesonephric remnants and neoplasms (7,10).

As aforementioned, due to the rarity and lack of high-quality evidences, no consensus exist regarding the best therapeutic method. Although radical hysterectomy with or without bilateral salpingo-oophorectomy and pelvic lymphadenectomy is the mainstay of treatment for early-stage disease, the efficacy of chemotherapy or radiotherapy is not clear, especially in locally advanced stages. More experiences on the management of this tumor would be necessary to achieve the best strategy (11). Currently, the treatment methods are adopted from guidelines for typical cervical adenocarcinoma. 

Prognosis of mesonephric carcinoma of cervix cannot be predicted accurately, due to the small number of reported cases with sufficient follow up. Majority of the cases have been diagnosed at stage I, but it seems that mesonephric carcinoma of cervix is not associated with a good prognosis. However, some believe that the mesonephric adenocarcinomas are more indolent malignancies compared to their Mullerian counterparts (12). Malignant mesonephric tumor of the cervix with initial manifestation of pulmonary metastasis has also been reported (13). The recurrence rate in a review study was reported as 32% and the mean recurrence interval was 24 months (1). Although majority of the cases are diagnosed at early stages, mesonephric carcinoma carries a poor prognosis. Our patient had a disappointing outcome with respect to her early and extensive relapse less than a year from completing adjuvant treatment.

Because of the deep location of the tumor in our case, lack of nuclear atypia and obvious mitotic activity, mesonephric hyperplasia was diagnosed on biopsy specimen; however, history of vaginal bleeding and cervical mass on physical examination were warning symptoms pointing out adenocarcinoma. Since accurate diagnosis is crucial for treatment planning and surgical strategy, pathologists and clinicians should be aware of this entity. Although our patient was in stage IB2, she did not receive optimal surgery. Despite adjuvant chemoradiotherapy, she developed peritoneal and locoregional recurrence after 9 months. 

## Conclusion 

As compared to the conventional cervical adenocarcinomas, mesonephric type appears to carry a more aggressive behavior that may require a more radical treatment approach.

## References

[1] Ditto A, Martinelli F, Bogani G, Gasparri ML, Donato VD, Paolini B (2016). Bulky mesonephric adenocarcinoma of the uterine cervix treated with neoadjuvant chemotherapy and radical surgery: report of the first case. Tumori..

[2] Abdul-Ghafar J, Chong Y, Han HD, Cha DS, Eom M (2013). Mesonephric Adenocarcinoma of the Uterine Cervix Associated with Florid Mesonephric Hyperplasia: A Case Report. J lifestyle Med..

[3] Dierickx A, Göker M, Braems G, Tummers P, Van den Broecke R (2016). Mesonephric adenocarcinoma of the cervix: Case report and literature review. Gynecol Oncol reports..

[4] Menon S, Kathuria K, Deodhar K, Kerkar R (2013). Mesonephric adenocarcinoma (endometrioid type) of endocervix with diffuse mesonephric hyperplasia involving cervical wall and myometrium: an unusual case report. Indian J Pathol Microbiol..

[5] Robboy SJ, Anderson MC, Russell P (2002). Pathology of the female reproductive tract.

[6] Erşahin Ç, Huang M, Potkul RK, Hammadeh R, Salhadar A (2005). Mesonephric adenocarcinoma of the vagina with a 3-year follow-up. Gynecol Oncol..

[7] Silver SA, Devouassoux-Shisheboran M, Mezzetti TP, Tavassoli FA (2001). Mesonephric adenocarcinomas of the uterine cervix: a study of 11 cases with immunohistochemical findings. Am J Surg Pathol..

[8] Tekin L, Yazici A, Akbaba E, Akin MN (2015). Mesonephric adenocarcinoma of the uterine cervix: A case report and review of the literature. J Pak Med Assoc..

[9] Kenny SL, McBride HA, Jamison J, McCluggage WG (2012). Mesonephric adenocarcinomas of the uterine cervix and corpus: HPV-negative neoplasms that are commonly PAX8, CA125, and HMGA2 positive and that may be immunoreactive with TTF1 and hepatocyte nuclear factor 1-β. Am J Surg Pathol..

[10] Ordi J, Nogales FF, Palacin A, Márquez M, Pahisa J, Vanrell JA (2001). Mesonephric adenocarcinoma of the uterine corpus: CD10 expression as evidence of mesonephric differentiation. Am J Surg Pathol..

[11] Puljiz M, Danolić D, Kostić L, Alvir I, Tomica D, Mamić I (2016). Mesonephric adenocarcinoma of endocervix with lobular mesonephric hyperplasia: case report. Acta Clin Croat..

[12] Brown S, MacNeil M, Talia K, Longano A (2013). A RARE CASE OF MESONEPHRIC ADENOCARCINOMA OF THE UTERINE CERVIX: A CASE REPORT. Pathol RCPA..

[13] Yeo MK, Choi SY, Kim M, Kim KH, Suh KS (2016). Malignant mesonephric tumor of the cervix with an initial manifestation as pulmonary metastasis: case report and review of the literature. Eur J Gynaecol Oncol..

